# A web-based dynamic nomogram for predicting readmission in patients with heart failure with preserved ejection fraction

**DOI:** 10.3389/fcvm.2025.1492717

**Published:** 2025-06-18

**Authors:** Yi Ji, Guodong Wang, Yue Hu, Xiaotong Wang, Wanling Wu, Yuanyuan Luo, Yanqing Pan, Jie Liu, Lei Li, Hong Zhu, Defeng Pan

**Affiliations:** ^1^ Department of Cardiology, The Affiliated Hospital of Xuzhou Medical University, Xuzhou, Jiangsu, China; ^2^ Cardiovascular Medicine Department, Beijing Bo’ai Hospital, China Rehabilitation Research Center, Capital Medical University, Beijing, China; ^3^ Department of General Practice, The Affiliated Hospital of Xuzhou Medical University, Xuzhou, Jiangsu, China

**Keywords:** dynamic nomogram, heart failure, readmission, predictive model, prognosis

## Abstract

**Background:**

The study aims to evaluate the efficacy of a web-based dynamic nomogram predicting the risk of heart failure (HF)-related rehospitalization within 1 year in patients with HF with preserved ejection fraction (HFpEF).

**Methods:**

The data of patients from two centers were categorized into training and test sets. Least absolute shrinkage and selection operator and multivariate logistic regression analysis were conducted on the training set data after selecting risk factors described in previous studies, and they were used to set up a nomogram. We then analyzed the area under the receiver operating characteristic curve (AUC-ROC) and calibration plot and conducted decision curve analysis (DCA) to confirm the efficacy of the nomogram.

**Results:**

The 1-year HF rehospitalization rates of patients with HFpEF were 23.7% and 22.8% in the two study centers, respectively. Age, body mass index, atrial fibrillation, triglyceride-glucose index, left ventricular ejection fraction, E/e, and angiotensin-converting enzyme inhibitors/angiotensin receptor blocker administration positively correlated with 1-year HF-related rehospitalization in patients with HFpEF. The dynamic nomogram was constructed based on the seven variables. The AUC-ROC of the training [0.801, 95% confidence interval (CI): 0.767–0.837] and the test datasets (0.773, 95% CI: 0.713–0.824) demonstrated that the model had good predictive ability for risk factors, the calibration plots demonstrated the excellent agreement. Additionally, the DCA curve showed that the model is highly effective with a threshold probability of 10%–80%.

**Conclusion:**

The dynamic nomogram model effectively predicts HF-related rehospitalization risk within 1 year in patients with HFpEF and helps determine high-risk categories among them.

## 1 Introduction

The prevalence of heart failure with preserved ejection fraction (HFpEF) has been increasing over the past several decades, despite advancements in the treatment and diagnosis of certain patient groups (1–3). In HFpEF, mechanisms involving diastolic dysfunction, myocardial fibrosis, and disturbances in calcium metabolism contribute to repeated hospitalizations. This increases medical costs and imposes a considerable burden on patients (4). The all-cause rehospitalization rate within 30 days in patients with HFpEF is 21%, and prior admissions for HF strongly indicate higher mortality and rehospitalization rates in HFpEF (5, 6). Therefore, early identification of high-risk groups of patients with HFpEF is helpful to improve their monitoring after discharge and reduce the possibility of HF-related rehospitalization.

Metabolic diseases, especially diabetes mellitus (DM), affect the incidence and prognosis of HF (7, 8). Additionally, insulin resistance has been closely associated with cardiac dysfunction (9). The triglyceride–glucose (TyG) index, known as a highly sensitive and reliable biomarker, reflects insulin resistance and has been indicative of HF prognosis (10–13). A substantial number of studies demonstrated that a higher TyG index denoted a higher risk of major cardiovascular adverse events, and the inclusion of TyG indices in HF prediction models reasonably improved the accuracy of identifying vulnerable patients with HF (13–16). Multiple clinical, metabolic, and hemodynamic factors have been linked to rehospitalization risk in patients with HFpEF. As a reliable prognostic predictor of cardiovascular disease, the TyG index combined with other risk factors can be more accurate in predicting the rehospitalization risk of patients with HFpEF.

While traditional nomograms are effective for visualizing fixed predictive models and quantifying risk based on a set of established factors, they are inherently staticAs such, they cannot be readily recalibrated or interactively adjusted when new patient data become available, nor can they easily accommodate evolving ranges of predictor values over time (17–19). A static nomogram provides only limited flexibility in representing continuous variables and may fail to accurately reflect the individualized risk profile when input values fall between predefined categories. Dynamic nomograms overcome these limitations by providing an interactive, web-based interface that continuously updates predicted probabilities as individual patient data are input, offering clinicians a more intuitive assessment tool (20). In this study, we aimed to develop a web-based dynamic nomogram that integrates the TyG index and other key variables to predict 1-year HF-related rehospitalization risk in HFpEF patients.

## 2 Materials and methods

### 2.1 Study population and data source

Data on the HF database of the Affiliated Hospital of Xuzhou Medical University and Zhongda Hospital were collected and retrospectively analyzed according to the 2021 European Society of Cardiology (ESC) Guidelines (1). This study recruited patients diagnosed with HFpEF from January 2018 to September 2021. Our research was performed as per the Declaration of Helsinki guidelines and approval from the Ethics Committee of the Affiliated Hospital of Xuzhou Medical University (ID: XYFY2022-KL465) and Southeast University School of Medicine (ZDSYLL066-P01).

The inclusion criteria were (1) HFpEF diagnoses following the 2021 ESC Guidelines which includes: A left ventricular ejection fraction (LVEF) ≥50%; Elevated natriuretic peptides (BNP or NT-proBNP); Evidence of left ventricular diastolic dysfunction (such as an abnormal E/e’ ratio or other markers of diastolic dysfunction, confirmed by echocardiography). Signs and symptoms of heart failure (dyspnea, fatigue, fluid retention, etc.) (1), (2) age of ≥18 years, and (3) the New York Heart Association classes II–IV of HF.

The exclusion criteria include (1) Patients with incomplete follow-up records (less than 12 months of follow-up (2) Patients with active malignancies, or any history of cancer that could interfere with heart failure management or rehospitalization outcomes, including recent cancer treatments. (3) Patients with more than 10% missing clinical data, particularly with missing information related to the primary outcomes (rehospitalization data, BMI measurements) (4). Patients with end-stage organ failure as defined by any of the following: End-stage renal disease: renal insufficiency receiving renal replacement therapy or with eGFR <15 ml/min/1.73 m^2^; End-stage liver disease: Child-Pugh score C or MELD score ≥15; Severe brain dysfunction with significant residual disability or Glasgow Coma Scale ≤8. (5) Patients with planned hospital admissions for non-urgent procedures, elective surgeries, or scheduled treatments unrelated to cardiovascular causes.

Among the 1,376 patients in the training cohort, 45 patients died without experiencing HF rehospitalization. Due to insufficient documentation, these patients were excluded from further analysis to ensure data accuracy. All clinical measurements, including laboratory results and echocardiographic measurements were collected within 24 h of hospital admission for HF.

### 2.2 Selection of pridictive model variables

Prior to building the model, we prepared the data by filling in missing values using multiple imputation and normalizing the features through standardization. We included various factors having potentially strong effect on rehospitalization by reviewing previous studies (21), revealing 54 variables covering demographic characteristics [age, body mass index (BMI), systolic blood pressure, diastolic blood pressure, and heart rate]; laboratory and functional results (glycated hemoglobin A, serum creatinine [Scr], triglyceride [TG], fast blood glucose [FBG], total cholesterol [TC], low-density lipoprotein cholesterol, N-terminal pro-brain natriuretic peptide [NT-proBNP], LVEF, left ventricular end-diastolic dimension, etc.); cardiovascular diseases and interventions in patient medical history along with others [hypertension, angina pectoris, coronary heart disease, myocardial infarction [MI], atrial fibrillation [AF], percutaneous coronary intervention, transient ischemic attack, peripheral artery disease, DM, etc.]; administration of medications [antiplatelet drugs, statins, diuretics, angiotensin-converting enzyme inhibitors [ACEI], angiotensin receptor blockers [ARB], beta-blockers, etc.]. The candidate variables are summarized and analyzed in Table 1. All patients had blood samples collected within 24 h of admission. Because NT-proBNP was right-skewed, it was log-transformed prior to analysis. The TyG index was determined as fasting TG level (mg/dl) × FBG level (mg/dl)/^2^. For variable selection, we first applied least absolute shrinkage and selection operator (LASSO) regression to the entire set of 54 candidate predictors. A *P*-value of <0.05 was considered statistically significant. LASSO approximates some coefficients to zero and enables the selection of a simpler model that excludes non-informative predictors by adding an L1 regular term. To identify the final seven varibles used in the nomogram, we constructed a multiple logistic regression model on the LASSO-selected variables. This process ensured that each variable retained in the final model provided significant predictive value while avoiding overfitting in the training set, there were 191 HF-related rehospitalization events. With 7 predictors in the final model, the events-per-variable (EPV) ratio was approximately 27 (191/7 ≈ 27.3), which is well above the commonly recommended threshold of 10 EPV. This indicates that our model's parameter estimates are likely stable and not unduly affected by overfitting.

**Table 1:** Baseline characteristics of the non-rehospitalization and rehospitalization groups.

Variables	Non-readmission group (*n* = 612)	Readmission group (*n* = 191)	*P-*value
Age	68.66 ± 12.85	74.50 ± 12.14	<0.001
BMI, kg/m^2^	24.80 (23.10, 27.20)	23.17 (21.19, 25.31)	<0.001
Drinking (*n*, %)			0.128
No	386 (63.1%)	132 (69.1%)	
Yes	226 (36.9%)	59 (30.9%)	
Smoking (*n*, %)			0.063
No	460 (75.2%)	156 (81.7%)	
Yes	152 (24.8%)	35 (18.3%)	
NYHA class (*n*, %)			0.246
II	76 (12.4%)	32 (16.8%)	
III	428 (69.9%)	123 (64.4%)	
IV	108 (17.6%)	36 (18.8%)	
Etiology of heart failure (*n*, %)			0.463
CHD	282 (46.1%)	98 (51.3%)	
Hypertension	84 (13.7%)	28 (14.7%)	
Heart valve diseases	26 (4.2%)	10 (5.2%)	
DCM	9 (1.5%)	3 (1.6%)	
Other	211 (34.5%)	52 (27.2%)	
Past medical history (*n*, %)			
Hypertension	325 (53.1%)	103 (53.9%)	0.842
Angina	147 (24.0%)	42 (22.0%)	0.564
Myocardial infarction	176 (28.8%)	40 (20.9%)	0.033
Coronary heart disease	360 (58.8%)	99 (51.8%)	0.088
AF	209 (34.2%)	123 (64.4%)	<0.001
PCI	160 (26.1%)	44 (23.0%)	0.389
Pacemaker	25 (4.1%)	13 (6.8%)	0.122
Stroke	139 (22.7%)	36 (18.8%)	0.259
TIA	64 (10.5%)	21 (11.0%)	0.833
PAD	16 (2.6%)	2 (1.0%)	0.201
DM	342 (55.88%)	121 (63.35%)	0.032
Dyslipidaemia	14 (2.3%)	2 (1.0%)	0.284
Asthma	3 (0.5%)	1 (0.5%)	0.954
COPD	26 (4.2%)	6 (3.1%)	0.495
OSAS	8 (1.3%)	2 (1.0%)	0.777
CKD	36 (5.9%)	14 (7.3%)	0.470
Thyroid dysfunctions	31 (5.1%)	5 (2.6%)	0.154
Anemia	41 (6.7%)	13 (6.8%)	0.959
Iron deficiency	173 (28.3%)	40 (20.9%)	0.045
SBP (mmHg)	132 (120, 146)	130 (120, 148.5)	0.733
DBP (mmHg)	80 (70, 87)	78 (70, 88.25)	0.578
HR	76 (68, 89)	75 (65, 88.25)	0.273
HbA1c	6.50 (5.80, 7.70)	6.70 (5.98, 8.30)	0.111
SCr (umol/L)	77.00 (60.00, 101.00)	80.50 (64.00, 108.50)	0.043
Cystatin C (mg/L)	1.81 (1.19, 2.48)	1.90 (1.32, 2.42)	0.498
Hb (g/L)	127 (109, 139)	119 (105.5, 132)	<0.001
Na (mmol/L)	140.00 (137.30, 142.20)	140.00 (137.88, 142.93)	0.410
K (mmol/L)	4.05 (3.72, 4.36)	4.10 (3.63, 4.44)	0.737
TG (mmol/L)	3.89 (3.19, 4.58)	3.87 (3.18, 4.74)	0.880
FBG (mmol/L)	5.63 (4.99, 7.21)	6.17 (5.24, 7.87)	0.010
TC (mmol/L)	1.31 (0.95, 1.90)	1.36 (1.01, 2.05)	0.066
TyG index	8.76 (8.40, 9.22)	8.85 (8.49, 9.37)	0.013
LDL (mmol/L)	2.42 (1.24, 3.20)	2.40 (1.67, 3.19)	0.117
UA (umo/L)	359.00 (283.00, 450.00)	372.50 (310.75, 501.25)	0.022
NT-proBNP (pg/ml)	1,834.00 (694.00, 4,215.04)	2,387.50 (911.00, 6,107.25)	0.015
LVEF (%)	56.0 (53.0, 63.0)	59.8 (56.0, 65.3)	<0.001
LVEDd	48.2 (40.2, 59.2)	51.1 (45.1, 58.1)	0.037
E/e’	11.36 (9.25, 14.62)	12.10 (9.81, 16.63)	0.013
Inotropic drugs (*n*, %)			
Diuretic	527 (86.1%)	164 (85.9%)	0.931
Nitrate esters	269 (44.0%)	82 (42.9%)	0.804
Digitonin	207 (33.8%)	75 (39.3%)	0.169
ACEI/ARB	551 (90.0%)	147 (77.0%)	<0.001
Beta-blockers	431 (70.4%)	153 (80.1%)	0.009
Aldosterone-receptor blocker	397 (64.9%)	139 (72.8%)	0.043
Antiplatelet drugs	446 (72.9%)	139 (72.8%)	0.978
Anticoagulant	172 (28.1%)	62 (32.5%)	0.247
Statin	465 (76.0%)	155 (81.2%)	0.137
CCB	117 (19.1%)	48 (25.1%)	0.073

BMI, body mass index; NYHA, New York Heart Association; CHD, coronary heart disease; DCM, dilated cardiomyopathy; AF, atrial fibrillation; PCI, percutaneous coronary intervention; TIA, transient ischemic attack; PAD, peripheral artery disease; DM, diabetes mellitus; COPD, chronic obstructive pulmonary disease; OSAS, obstructive sleep apnea syndrome; CKD, chronic kidney disease; SBP, systolic blood pressure; DBP, diastolic blood pressure; HR, heart rate; HbA1c, glycated hemoglobin A; Scr: serum creatinine; Hb, hemoglobin; Na, Natrium; K, Kalium; TG, triglyceride; FBG, fast blood glucose; TC, total cholesterol; TyG, triglyceride-glucose; LDL-C, low-density lipoprotein cholesterol; UA, uric acid; NT-proBNP, N-terminal pro brain natriuretic peptide; LVEF, left ventricular ejection fraction; LVEDd, left ventricular end-diastolic dimension; ACEI, angiotensin-converting enzyme inhibitor; ARB, angiotensin receptor blockers; CCB, calcium channel blocker.

### 2.3 Follow-up of patients

The enrolled patients with HFpEF were followed up after 1 year, and their characteristics were retrospectively analyzed after the conducted diagnostics. The follow-up method included electronic medical record system inquiries and outpatient visits, supplemented by telephone follow-ups and home visits as required. The focus of the follow-up was on the patient's HF-related rehospitalization. The final follow-up date was September 30, 2022. The endpoint event was HF-related rehospitalization occurring within one year after the discharge of patients with HFpEF.

### 2.4 Statistical analysis

Statistical Package for the Social Sciences version 22.0 was applied for data description and intergroup comparison. Shapiro–Wilk test was employed to verify the normal distribution of the analyzed data. Descriptive statistics were presented as mean ± standard deviation (X ± SD), and a *t*-test was applied to comparatively analyze intergroup differences. Median (M) and interquartile range (M [P25, P75]) were used for non-normally distributed data, and the comparison of differences between groups was achieved by non-parametric tests. Frequencies and percentages (%) were described for categorical data, and a chi-square test was used for its comparison between the studied groups. Least absolute shrinkage and selection operator (LASSO) regression and multiple logistic regression analysis were conducted to estimate the relative risk and corresponding 95% confidential interval (CI) for each variable. As described above, LASSO was used for initial variable selection, followed by backward stepwise elimination in the multivariate logistic regression model to finalize the predictors. R version 3.6.4 was used to develop the predictive model, and a web-based dynamic nomogram was established. The area under the receiver operating characteristic (AUC–ROC) curve was used to assess its discriminative capability. The calibration ability of it was analyzed via a calibration curve, and its clinical value was evaluated by DCA curve.

## 3 Results

### 3.1 Baseline characteristics

**Figure 1:**
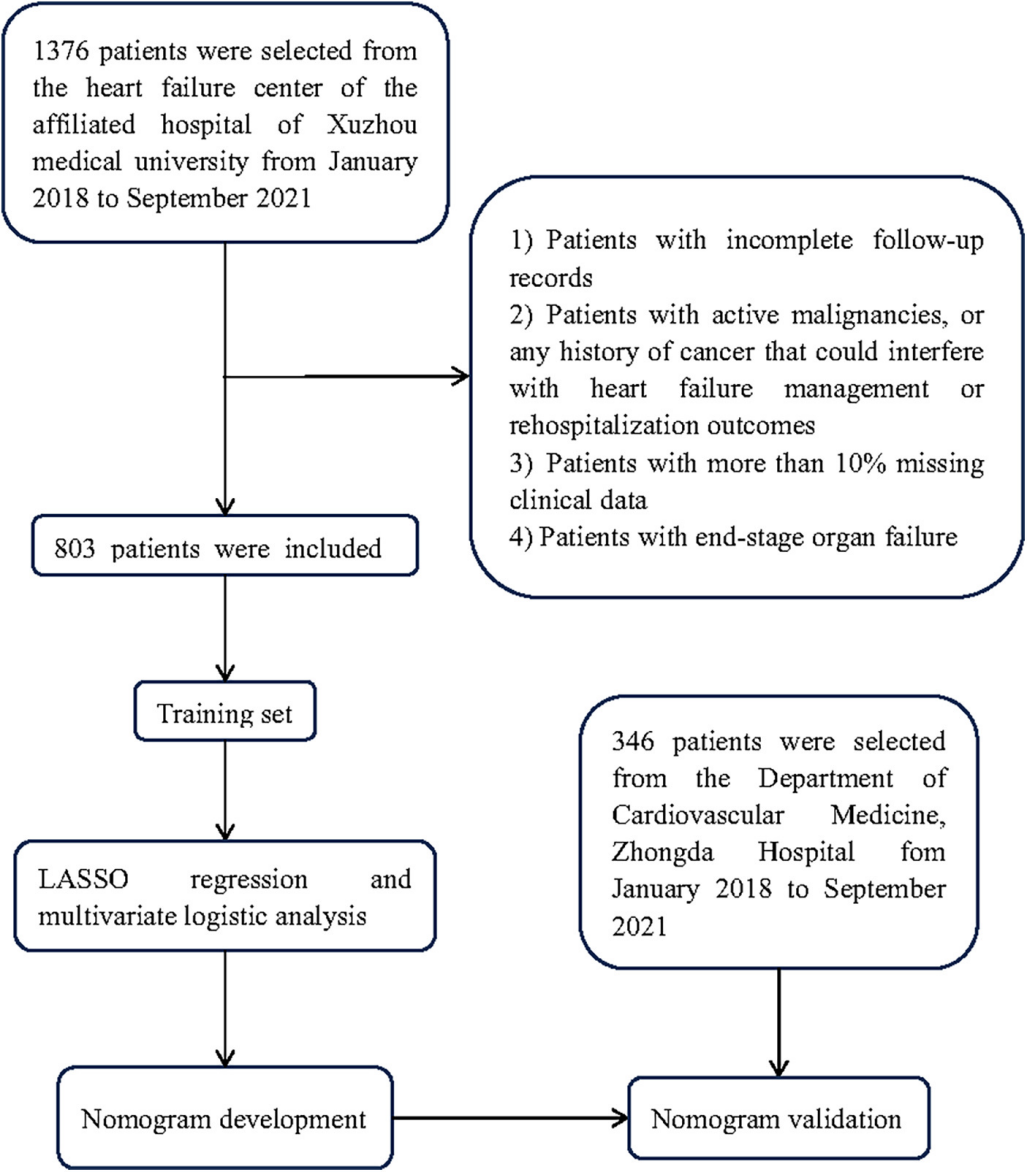
Flowchart of patient selection performed in this study.

The data of 1,376 patients with HFpEF were extracted from the Heart Failure Center database of Xuzhou Medical University Affiliated Hospital from January 2018 to September 2021, and that of 756 from Zhongda Hospital were obtained. Eventually, the training set included 803 patients from the Affiliated Hospital of Xuzhou Medical University, and the validation set enrolled 346 from Zhongda Hospital. Figure 1 outlines the flowchart of inclusion/exclusion criteria**.** Patients were assigned to the rehospitalization (*n* = 191) and non-rehospitalization groups (*n* = 612) according to their rehospitalization records. By comparison, we revealed that the latter was characterized by younger age, higher BMI, higher rate of MI, but lower DM and AF incidence, a higher iron deficiency rate, higher hemoglobin and lower SCr levels, lower FBG, TyG index, NT-proBNP, UA, LVEF, and E/e values, and higher administration rates of ACEI/ARB, beta-blockers, and aldosterone receptor blockers.

### 3.2 LASSO and multivariate logistic regression analysis of HF rehospitalization risk in patients with HFpEF

**Figure 2:**
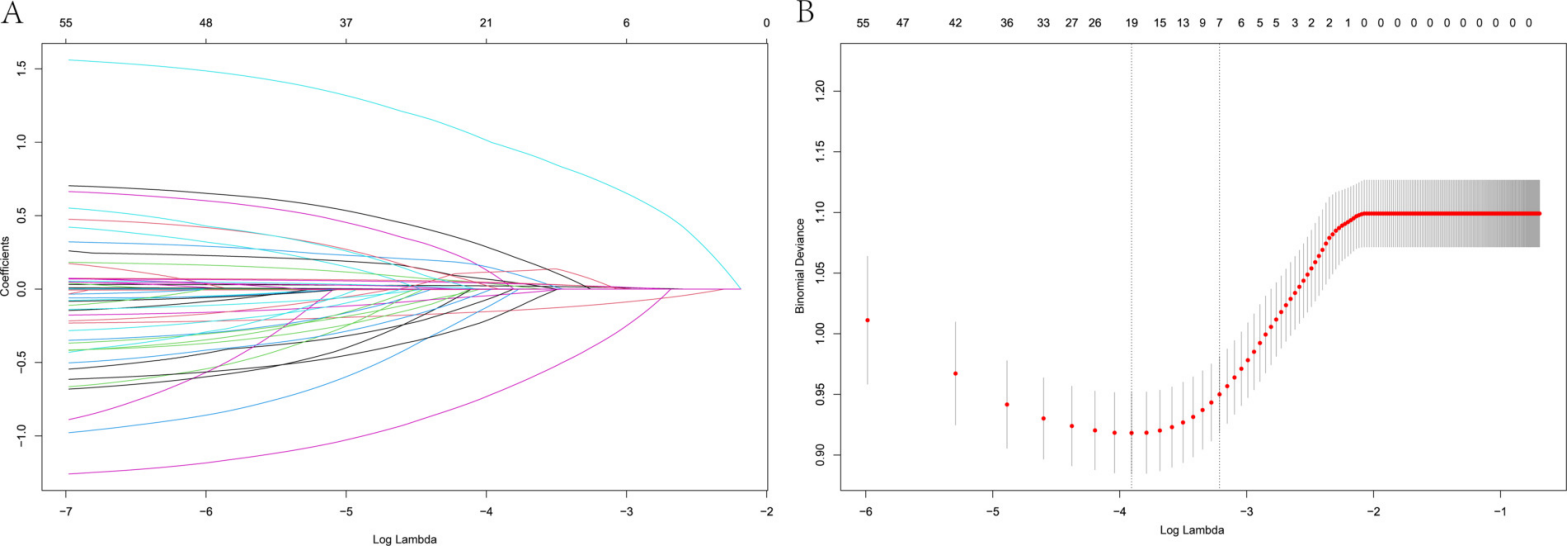
LASSO regression model showing predictors of 1-year HF rehospitalization. **(A)** LASSO regression model cross-validation plot. A vertical line represents the optimum with the minimum criterion. With the optimal *λ* = 0.0402, seven variables were selected for further analysis. **(B)** Coefficient profile plot of predictors.

The LASSO analysis revealed that the risk factors of HF-related rehospitalization in patients with HFpEF were age, BMI, AF, TyG index, LVEF, E/e, and ACEI/ARB administration (Figure 2). The above seven variables were then covered by the multivariate logistic regression analysis, revealing age [odds ratio (OR): 1.036; 95% CI: 1.019–1.053], BMI (OR: 0.805; CI: 0.754–0.859), AF (OR: 3.565; CI: 2.435–5.219), TyG (OR:1.606; CI: 1.199–2.153), LVEF (OR: 1.062; CI: 1.033–1.092), E/e (OR: 1.084; CI: 1.040–1.129), and ACEI/ARB administration (OR: 0.359, CI: 0.219–0.587) (Table 2).

**Table 2:** Multivariate logistic analysis for 1-year rehospitalization in patients with HFpEF.

Variables	*β*	s*χ*	Standardized effect	Waldχ2	OR (95% CI)	*P*-value
			Size (β/sχ)			
Age, years	0.035	0.008	4.375	17.588	1.036 (1.019, 1.053)	<0.001
BMI, kg/m^2^	−0.217	0.033	−6.576	42.791	0.805 (0.754, 0.859)	<0.001
AF	1.271	0.194	6.552	42.725	3.565 (2.435, 5.219)	<0.001
TYG	0.474	0.149	3.181	10.063	1.606 (1.199, 2.153)	0.002
LVEF, %	0.06	0.014	4.286	18.539	1.062 (1.033, 1.092)	<0.001
E/e	0.08	0.021	3.81	14.619	1.084 (1.040, 1.129)	<0.001
ACEI/ARB	−1.025	0.252	−4.067	16.592	0.359 (0.219, 0.587)	<0.001

BMI, body mass index; AF, atrial fibrillation; TyG, triglyceride-glucose; LVEF, left ventricular ejection fraction; ACEI, angiotensin-converting enzyme inhibitor; ARB, angiotensin receptor blockers; β, effect size; sχ, standard error; (β/sχ): standardized effect size.

### 3.3 Comparison of the training and test datasets

The 30-day HF-related rehospitalization rates in the training and test datasets were 17.8% and 17.9%, and the 1-year HF rehospitalization rates composed 23.7% and 22.8%, respectively. Except for COPD history, no statistical differences were detected between the sets (Table 3).

**Table 3:** Comparison of training and test datasets.

Variables	Training set (*n* = 803)	Validation set (*n* = 346)	*P*-value
Age	70.05 ± 12.92	68.83 ± 12.60	0.172
BMI, kg/m^2^	24.24 (22.64, 26.70)	24.90 (22.88, 27.20)	0.141
Drinking (*n*, %)			0.585
No	518 (64.5%)	229 (66.2%)	
Yes	285 (35.5%)	117 (33.8%)	
Smoking (*n*, %)			0.108
No	616 (76.7%)	250 (72.3%)	
Yes	187 (23.3%)	96 (27.7%)	
NYHA class (*n*, %)			0.176
II	108 (13.4%)	47 (13.6%)	
III	551 (68.6%)	221 (63.9%)	
IV	144 (17.9%)	78 (22.5%)	
Etiology of heart failure (*n*, %)			0.576
CHD	380 (47.3%)	168 (48.6%)	
Hypertension	112 (13.9%)	50 (14.5%)	
Heart valve diseases	36 (4.5%)	21 (6.1%)	
DCM	12 (1.5%)	7 (2.0%)	
Other	263 (32.8%)	100 (28.9%)	
Past medical history (*n*, %)			
Hypertension	428 (53.3%)	179 (51.7%)	0.626
Angina	189 (23.5%)	82 (23.7%)	0.952
Myocardial infarction	216 (26.9%)	82 (23.7%)	0.256
Coronary heart disease	459 (57.2%)	200 (57.8%)	0.840
AF	332 (41.3%)	125 (36.1%)	0.097
PCI	204 (25.4%)	96 (27.7%)	0.407
Pacemaker	38 (4.7%)	21 (6.1%)	0.346
Stroke	175 (21.8%)	71 (20.5%)	0.629
TIA	85 (10.6%)	37 (10.7%)	0.956
PAD	18 (2.2%)	5 (1.4%)	0.377
DM	463 (57.66)	213 (61.56)	0.218
Dyslipidaemia	16 (2.0%)	8 (2.3%)	0.728
Asthma	4 (0.5%)	1 (0.3%)	0.621
COPD	32 (4.0%)	24 (6.9%)	0.033
OSAS	10 (1.2%)	4 (1.2%)	0.899
CKD	50 (6.2%)	12 (3.5%)	0.058
Thyroid dysfunctions	36 (4.5%)	8 (2.3%)	0.079
Anemia	54 (6.7%)	20 (5.8%)	0.550
Iron deficiency	213 (26.5%)	80 (23.1%)	0.225
SBP (mmHg)	131 (120, 147)	130 (120, 147)	0.287
DBP (mmHg)	80 (70, 87)	79 (70, 88.25)	0.864
HR	76 (67, 89)	78 (69, 90)	0.126
HbA1c	6.60 (5.90, 7.90)	6.50 (5.80, 7.73)	0.350
SCr (umol/L)	78 (61, 103)	78 (63, 96)	0.688
Cystatin C (mg/L)	1.82 (1.21, 2.45)	1.89 (1.17, 2.61)	0.556
Hb (g/L)	125 (108, 138)	126.5 (112, 140)	0.077
Na (mmol/L)	140 (137.3, 142.3)	140.25 (138, 142.7)	0.071
K (mmol/L)	4.06 (3.69, 4.38)	4.06 (3.75, 4.40)	0.704
TG (mmol/L)	3.89 (3.18, 4.64)	3.85 (3.21, 4.63)	0.968
FBG (mmol/L)	5.75 (5.03, 7.31)	5.57 (5.05, 7.40)	0.436
TC (mmol/L)	1.33 (0.97, 1.94)	1.34 (0.99, 1.91)	0.733
TyG index	8.78 (8.42, 9.25)	8.79 (8.41, 9.22)	0.848
LDL (mmol/L)	2.42 (1.33, 3.20)	2.40 (1.28, 3.18)	0.986
UA (umo/L)	362 (291, 457)	361 (287, 456)	0.525
NT-proBNP (pg/ml)	1,969 (749. 8,4,321)	1,576.5 (717.64, 3,667.03)	0.078
LVEF (%)	56.7 (53.6, 63.3)	56.0 (52.0, 62.0)	0.865
LVEDd	49.7 (40.2, 58.6)	52.0 (43.0, 60.0)	0.765
E/e’	11.44 (9.33, 15.06)	10.92 (8.87, 14.41)	0.081
Inotropic drugs (*n*, %)			
Diuretic	691 (86.1%)	291 (84.1%)	0.390
Nitrate esters	351 (43.7%)	153 (44.2%)	0.873
Digitonin	282 (35.1%)	113 (32.7%)	0.421
ACEI/ARB	698 (86.9%)	297 (85.8%)	0.620
Beta-blockers	584 (72.7%)	254 (73.4%)	0.811
Aldosterone-receptor blocker	536 (66.7%)	221 (63.9%)	0.345
Antiplatelet Drugs	585 (72.9%)	254 (73.4%)	0.845
Anticoagulant	234 (29.1%)	100 (28.9%)	0.935
Statin	620 (77.2%)	266 (76.9%)	0.902
CCB	165 (20.5%)	64 (18.5%)	0.425

BMI, body mass index; NYHA, New York Heart Association; CHD, coronary heart disease; DCM, dilated cardiomyopathy; AF, atrial fibrillation; PCI, percutaneous coronary intervention; TIA, transient ischemic attack; PAD, peripheral artery disease; DM, diabetes mellitus; COPD, chronic obstructive pulmonary disease; OSAS, obstructive sleep apnea syndrome; CKD, chronic kidney disease; SBP, systolic blood pressure; DBP, diastolic blood pressure; HR, heart rate; HbA1c, glycated hemoglobin A; Scr, serum creatinine; Hb, hemoglobin; Na, Natrium; K, Kalium; TG, triglyceride; FBG, fast blood glucose; TC, total cholesterol; TyG, triglyceride-glucose; LDL-C, low-density lipoprotein cholesterol; UA, uric acid; NT-proBNP, N-terminal pro brain natriuretic peptide; LVEF, left ventricular ejection fraction; LVEDd, left ventricular end-diastolic dimension; ACEI, angiotensin-converting enzyme inhibitor; ARB, angiotensin receptor blockers; CCB, calcium channel blocker.

### 3.4 Development and validation of a dynamic nomogram

**Figure 3:**
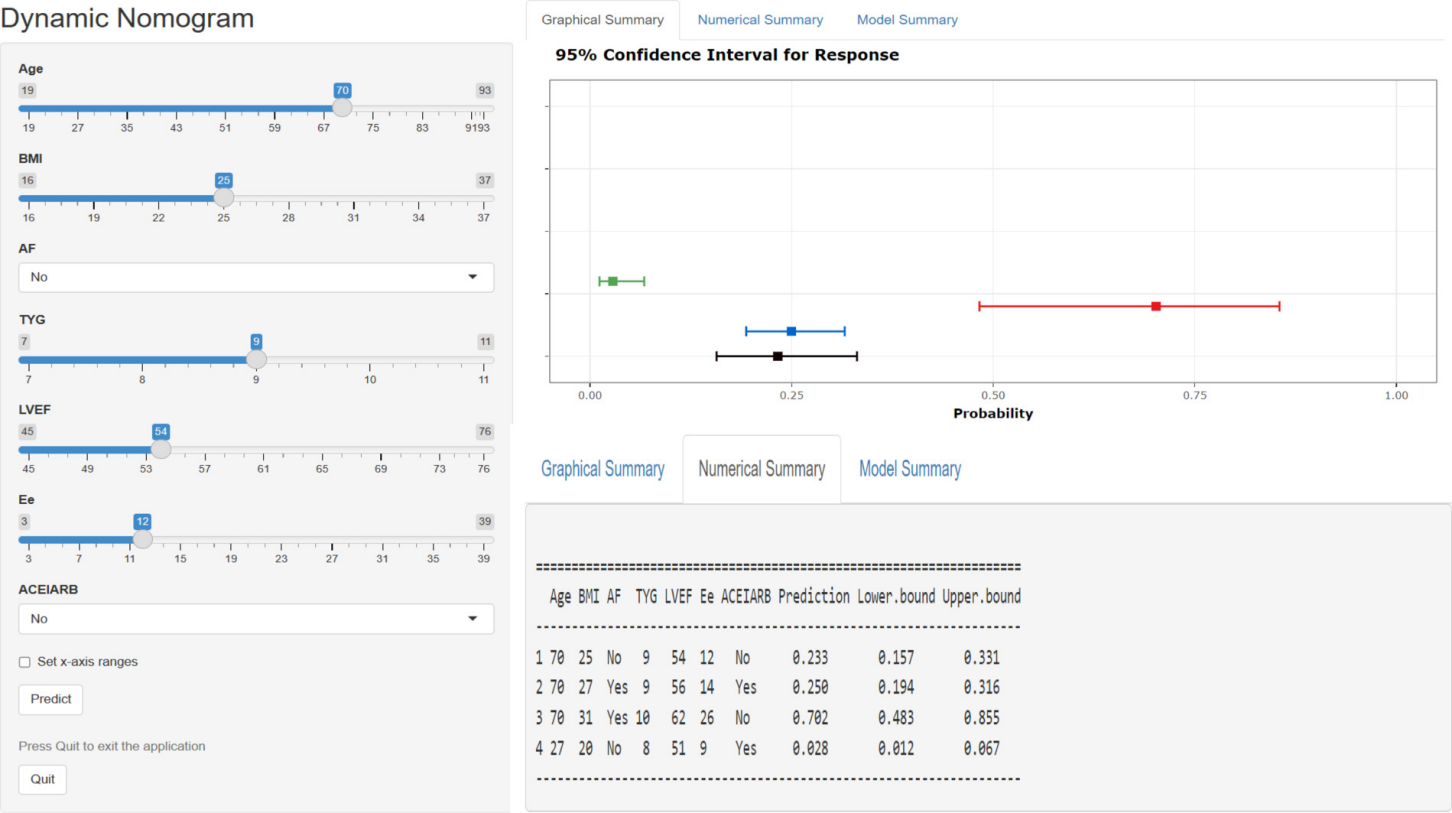
Web-based dynamic nomogram applied for predicting 1-year rehospitalization in patients with hFpEF. The input characteristics include age, BMI, AF, TyG index, LVEF, E/e, and ACEI/ARB administration, and they can be entered at https://hfpef.shinyapps.io/hfpef/, where a user can get the corresponding probability of 1-year rehospitalization. **(A)** Input page: enter a patient's information following the appropriate variables on this page. **(B)** Graphical summary: this page reveals the probability of a patient being rehospitalized with HF with a 95% confidence interval. **(C)** Numerical summary: display the specific values of the markers and predicted outcomes. HFpEF, heart failure with preserved ejection fraction; BMI, body mass index; AF, atrial fibrillation; TyG, triglyceride–glucose; LVEF, left ventricular ejection fraction; ACEI, angiotensin-converting enzyme inhibitor; ARB, angiotensin receptor blockers.

**Figure 4:**
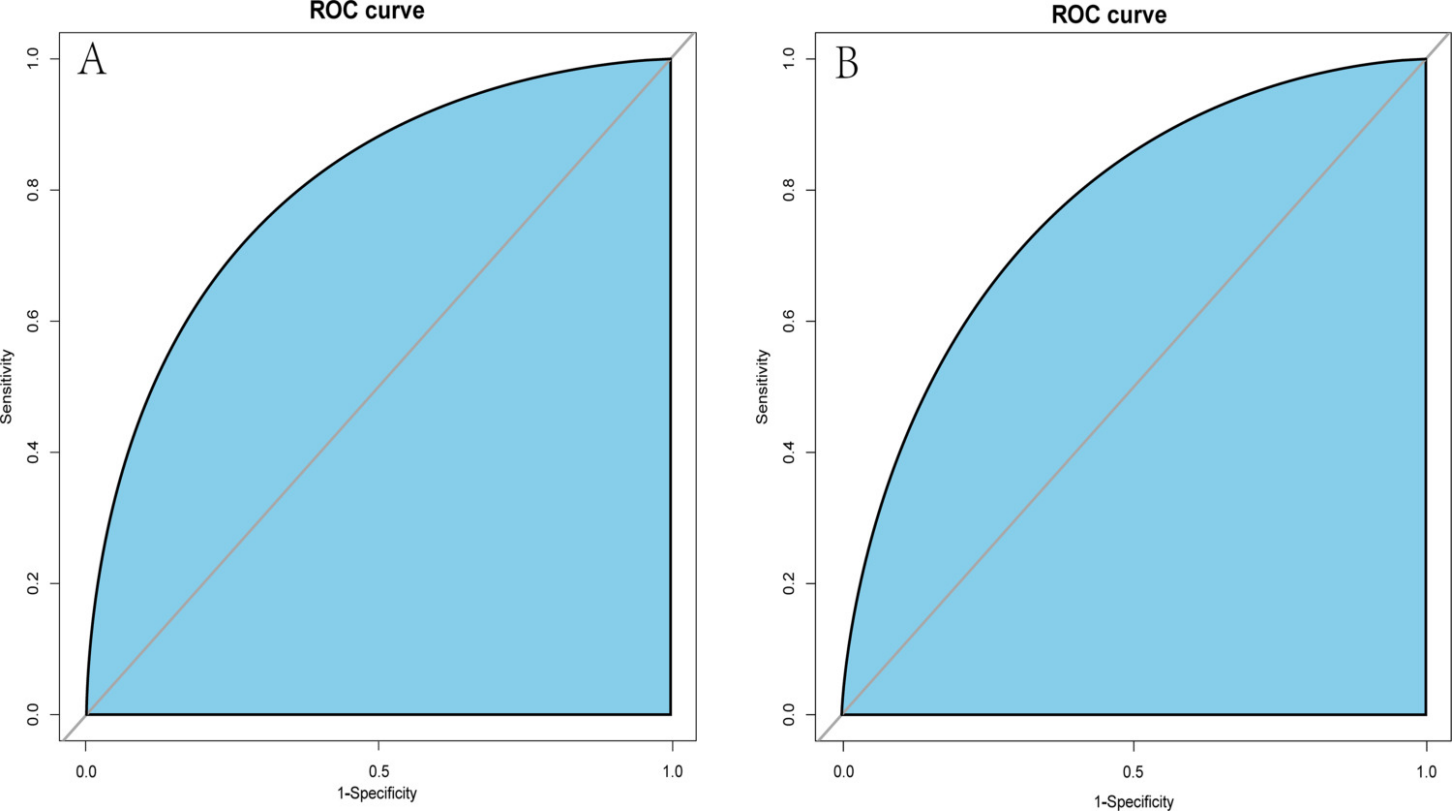
ROC curves of the dynamic nomogram based on the data of the training **(A)** and test datasets **(B)**. AUC–ROC, the area under the receiver operating characteristic.

**Figure 5:**
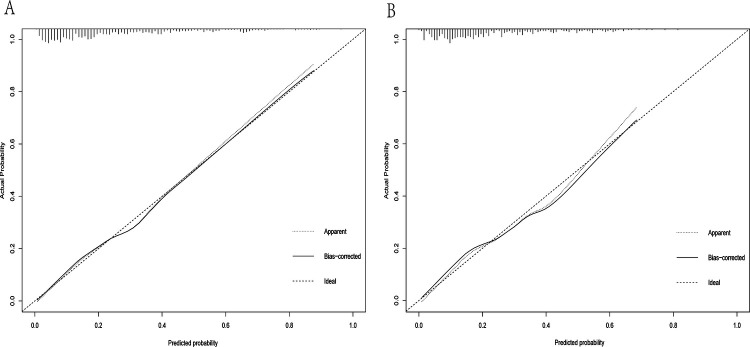
Calibration curve of the dynamic nomogram based on the data of the training **(A)** and test datasets **(B)**.

**Figure 6:**
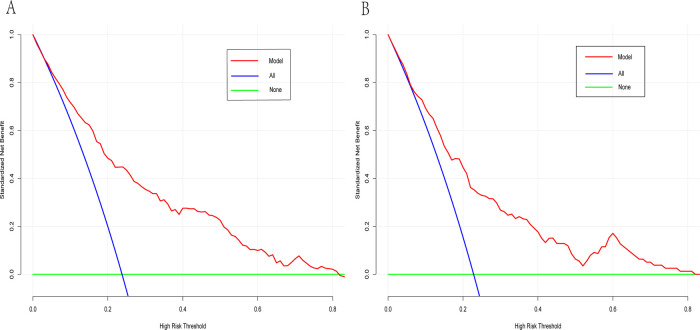
Decision curve analysis curve of the dynamic nomogram having the data of training **(A)** and test datasets **(B)**.

A dynamic nomogram was constructed based on the seven independent predictors identified in the multivariate analysis (Table 2). To facilitate clinical application, the model was deployed as an interactive web-based tool using the R Shiny framework. The nomogram is freely accessible online at https://hfpef.shinyapps.io/hfpef/, where clinicians can input individual patient variables to instantly estimate the 1-year risk of HF-related rehospitalization for patients with HFpEF (Figure 3). The nomogram model demonstrated good discriminative performance, with an AUC-ROC of 0.801 (95% CI: 0.767–0.837) in the training set and 0.773 (95% CI: 0.713–0.824) in the external validation set (Figure 4). In addition, the calibration curve showed strong agreement between predicted and observed outcomes (Figure 5), and decision curve analysis (DCA) demonstrated favorable net clinical benefit across a range of threshold probabilities from 10% to 80% (Figure 6). Our model (red line) provides greater benefit than the other two strategies (treating all or no patients) across most risk thresholds (10%–80%), both in the training (A) and validation datasets (B). This confirms that our nomogram is clinically useful for predicting HFpEF rehospitalization.

## 4 Discussion

HF manifests itself as a complex clinical syndrome, and HFpEF has been recognized as one of its primary forms. Since rehospitalization has become one of the crucial problems in patients with HF (22), establishing a model to identify the risk of readmission is necessary. Such a model could help prevent readmissions and enhance the overall management of HFpEF patients. In this study, age, BMI, AF, TyG index, LVEF, E/e, and the presence of ACEI/ ARB therapy in medical history were strongly associated with 1-year rehospitalization risk in patients with HFpEF. Based on these variables, we established a dynamic nomogram that, after external validation, effectively predicts 1-year rehospitalization risk in the mentioned category.

The incidence of HFpEF has been increasing in recent years, as the global population tends to age. Frailty, reduced endurance, and frequent rehospitalizations have become more prevalent with the aging of patients with HFpEF (23, 24). Our study revealed that 1-year rehospitalization risk of patients with HFpEF is not very high, which may be explained by the older average age of the analyzed population. A study in 2017 revealed the largest number of older adults having HFpEF in Europe (25). Post-discharge health education and follow-up should be further tailored for this patient group.

Obesity contributes to coronary microvascular dysfunction and is a significant risk factor for the onset and progression of HFpEF (26, 27). Paradoxically, the “obesity paradox” observed in both HFpEF and HFrEF suggests that, although obesity increases cardiovascular risk, higher BMI may confer a survival advantage once heart failure is established (28, 29). Several mechanisms may underlie this phenomenon. Adipose tissue functions as an active endocrine organ, secreting adipokines such as leptin and adiponectin, which have anti-inflammatory properties that may mitigate the chronic inflammation characteristic of HFpEF (30). Additionally, heart failure is often associated with a chronic catabolic state leading to muscle loss, higher BMI may reflect greater muscle mass and reduced susceptibility to muscle wasting (31). Greater metabolic reserves could help patients better withstand HF-related physiological stress (28). Our findings support a protective role of elevated BMI against HF-related rehospitalization. Similarly, Mulmi et al. reported an inverse relationship between BMI and rehospitalization rates in HF, reinforcing this protective association (32). Padwal et al. further confirmed the presence of the obesity paradox in patients with both preserved and reduced ejection fraction, aligning with our results (33). AF exhibits a high incidence in HFpEF, because these conditions share similar risk factors (34), and patients with AF in HFpEF frequently have poorer prognosis (35). Our research revealed that multivariate logistic regression analysis demonstrated AF as a significantly higher risk factor than others, which again confirmed the importance of AF management in patients with HFpEF. Alcohol withdrawal and healthy weight loss should be recommended for patients with AF, whereas ventricular rhythm should be controlled, and HF drugs that potentially interfere with AF should be avoided (36).

The TyG index is frequently used as a simple surrogate marker for insulin resistance. Patients with a high TyG index frequently demonstrate metabolic abnormalities, such as DM and dyslipidemia. Additionally, a high TyG index is associated with chronic inflammation, as insulin resistance induces adipose tissue to secrete pro-inflammatory cytokines, including interleukin-6 and tumor necrosis factor-α. These inflammatory mediators cause damage and fibrosis in myocardial cells, thereby exacerbating HF progression (37). As a relatively new indicator, the TyG index has been closely associated with cardiovascular disease prognoses. Recent research revealed that patients with HFpEF had higher values of the TyG index (38). These patients' condition is frequently accompanied by DM and dyslipidemia; thus, hypoglycemic and lipid-lowering drugs may affect the TyG index calculation. This finding further supports that effective blood glucose and lipid management reduces the risk of rehospitalization in patients with HFpEF.

Color Doppler echocardiography is a simple and effective method for HF diagnostics at present. LVEF visually displays left ventricular ejection capacity, whereas E/e reflects left ventricular diastolic function (39). LVEF reduction is not obvious in patients with HFpEF, but our analysis reveals that it remains an independent risk factor for 1-year rehospitalization. Additionally, left ventricular diastolic function is a key diagnostic feature of HFpEF. Therefore, an improvement in cardiac function is necessary, even if the symptoms and signs of HF do not clinically manifest in patients. Delayed left ventricular remodeling is an essential goal in HF treatment, and ACEI and ARB are predominantly administered for this purpose and are therefore widely applied in treating various HF types (40). Interestingly, our results indicated that a higher LVEF was associated with an increased risk of HF rehospitalization. This finding is counterintuitive, as it is typically expected that lower LVEF would indicate more severe heart failure and therefore a higher risk of readmission. We believe this unexpected result may partly be explained by competing risks, where patients with lower LVEF may have a higher risk of death, thus reducing the opportunity to experience rehospitalization. Our study reveals that ACEI/ARBs significantly correlate with 1-year rehospitalization risk in patients with HFpEF compared with diuretics and aldosterone receptor antagonists. Additionally, ACEI/ARBs still reduce the risk of rehospitalizations in patients without contraindications.

In this study, we developed a web-based dynamic risk calculator that offers a convenient and interactive way to assess individualized risk by allowing real-time patient data input and immediate visualization of the predicted 1-year HF-related rehospitalization probability. To further promote clinical implementation, the tool is accessible online (https://hfpef.shinyapps.io/hfpef/). Clinicians can input routinely available parameters-such as age, BMI, AF status, TyG index, LVEF, E/e, and ACEI/ARB use-and receive intuitive graphical and numerical outputs. This supports timely clinical decision-making regarding therapeutic adjustment and risk communication with patients. External validation of the model confirmed its robustness and applicability, highlighting its potential as a practical tool in routine HFpEF management. For illustrative purposes, a real-world example is provided: a 60-year-old patient with atrial fibrillation, a BMI of 24 kg/m^2^, a TyG index of 9, LVEF of 53%, an E/e ratio of 17, and prior ACEI/ARB therapy. The predicted risk of rehospitalization in this case was 11.82% and the actual outcome was that the patient did not experience rehospitalization.

To address the risk of model overfitting, our analysis yielded an EPV ratio of approximately 27 (191 events for 7 predictors), which exceeds the conventional threshold of 10 EPV and reduces the likelihood of statistical overfitting. We employed a two-step variable selection procedure involving LASSO regression and multivariate logistic modeling, which further mitigates the risk of overfitting by shrinking coefficients and removing non-informative variables. The model also demonstrated strong generalization performance in an independent external dataset (AUC = 0.773), supporting its robustness. To improve the model's generalizability and reliability, future research should include external validation in ethnically and geographically diverse populations. Multicenter, prospective studies across various healthcare systems are crucial to confirm the predictive value of identified risk factors. These efforts will enhance the nomogram's clinical utility and support the development of decision tools, promoting timely risk stratification and targeted management in HFpEF. Furthermore, recent literature has emphasized the importance of classifying HF patients into four hemodynamic phenotypes: (1) warm and dry, (2) warm and wet, (3) cold and wet, and (4) cold and dry. Particularly, the “cold and dry” phenotype has been independently linked to worse clinical outcomes among elderly HFpEF patients. Therefore, incorporating hemodynamic phenotyping into future predictive models could further improve individualized risk stratification and clinical decision-making in HFpEF patients.

## 5 Limitations

Some limitations of the study should be addressed. First, the sample size is relatively small, which requires more data verification, and more studies are warranted to further validate the reliability and applicability of our nomogram. Moreover, the research did not cover the information on the use of SGLT2 inhibitor therapy among patients, which typically provides significant cardioprotective benefits in those with HF. The absence of the analysis of these data may affect the general applicability of our nomogram. Additionally, the external validity of the model is limited because only hospitals in China were selected for patient recruitment. In review and both analyzed cohorts within the model were limited to the Asian population. Finally, A limitation of our study is the lack of time-of-death data for patients, which precluded us from conducting a proper competing risk analysis. Therefore, we were unable to account for the potential bias introduced by patients who died before experiencing HF readmission. We encourage further research that includes detailed time-to-event data to more comprehensively assess the impact of competing risks (41, 42).

## 6 Conclusion

We developed and validated an interactive web-based nomogram that predicts 1-year rehospitalization risk in HFpEF patients using routinely collected clinical data. Unlike existing models focused on mortality or HFrEF, our tool specifically targets HFpEF rehospitalization. It can assist clinicians in tailoring treatment plans according to patients' rehospitalization risk.

## Data Availability

The raw data supporting the conclusions of this article will be made available by the authors, without undue reservation.
